# Orthodontic System Modeled and Simulated with the Lingual Technique to Assess Tooth Forces

**DOI:** 10.3390/diagnostics14111171

**Published:** 2024-05-31

**Authors:** Abbas Hazem, Felicia Ileana Mărășescu, Mihaela Jana Țuculină, Alexandru Dan Popescu, Dragoș Laurențiu Popa, Lelia Laurența Mihai, Cristian Niky Cumpătă, Alexandru Iliescu, Petre Mărășescu, Ionela Teodora Dascălu

**Affiliations:** 1 Department of Orthodontics, Faculty of Dental Medicine, University of Medicine and Pharmacy of Craiova, 200349 Craiova, Romania; hazem6070@gmail.com (A.H.); ciuca_felicia@yahoo.com (F.I.M.); marceldascalu@yahoo.com (I.T.D.); 2 Department of Endodontics, Faculty of Dental Medicine, University of Medicine and Pharmacy of Craiova, 200349 Craiova, Romania; mtuculina@yahoo.com (M.J.Ț.); alexandrudanpopescu20@gmail.com (A.D.P.); 3 Department of Automotive, Transportation and Industrial Engineering, Faculty of Mechanics, University of Craiova, 200478 Craiova, Romania; popadragoslaurentiu@yahoo.com; 4 Department of Periodontology, Faculty of Dental Medicine, University Titu Maiorescu of Bucharest, 031593 Bucharest, Romania; lelia_mihai2000@yahoo.com; 5 Department of Oromaxillofacial Surgery, Faculty of Dental Medicine, University Titu Maiorescu of Bucharest, 031593 Bucharest, Romania; 6 Department of Oral Rehabilitation, Faculty of Dental Medicine, University of Medicine and Pharmacy of Craiova, 200349 Craiova, Romania; dentalexro@gmail.com; 7 Department of Dental Prothesis Tehnology, Faculty of Dental Medicine, University of Medicine and Pharmacy of Craiova, 200349 Craiova, Romania

**Keywords:** cone beam computed tomography, finite element method, lingual appliance, virtual model, forces

## Abstract

CBCT (cone beam computed tomography) is an imaging investigation that provides three-dimensional (3D) images of craniofacial structures. The purpose of this study is to determine the mechanical behavior of an orthodontic system where the lingual treatment technique was used in a 25-year-old female patient from whom a set of CBCT scans was used. CBCT images were processed through software programs such as Invesalius, Geomagic, and Solid Works, to create models containing virtual solids. These models were then imported into Ansys Workbench 2019 R3 (a finite element method software program) for successive simulations to generate displacement maps, deformations, stress distributions, and diagrams. We observed that in the lingual technique, the lowest force occurring on the maxillary teeth is at 1.1, while the highest force appears at 2.3. In the mandible, the lowest force occurs at 4.6, and the highest force at 3.1. The values of the forces and the results of the finite element method can represent a basis for the innovation of new orthodontic springs and also of bracket elements. Thus, by using new technologies, orthodontic practice can be significantly improved for the benefit of patients. Other virtual methods and techniques can be used in future studies, including the application of virtual reality for orthodontic diagnosis.

## 1. Introduction

The WHO appreciates that dento-maxillary anomalies represent a significant global oral health issue, having implications on both the aesthetics and normal functionality of the dento-maxillary apparatus. Orthodontic treatment corrects these issues and thus contributes to increasing the quality of life of patients [1].

In addition to its careful planning, effective anchorage management is also necessary for the success of orthodontic treatment. For orthodontists, a major problem in orthodontics over time has been anchorage. Modern anchorage techniques and devices, including orthodontic mini-implants, have facilitated improved anchorage. Their use has become essential in orthodontic treatment, making it possible to solve some difficult cases [2].

Currently, two-dimensional imaging techniques are widely used by orthodontists. The fact that these techniques do not provide precise data about the depth of the structures represents their disadvantage [2].

Along with other investigations, the implementation of CBCT in orthodontics has led to the rapid and correct establishment of orthodontic diagnosis. CBCT provides three-dimensional (3D) images of craniofacial structures. It also helps in the quick and definitive determination of orthodontic diagnosis, treatment planning, and the assessment of treatment outcomes. Additionally, CBCT allows for careful monitoring of the patient throughout the orthodontic treatment process. In orthodontics, it is successfully used in cases with anomalies of teeth position for visualizing teeth in their entirety in three-dimensional images [3,4,5].

The increasing addressability of patients with various dento-maxillary anomalies to orthodontist specialists has also increased their requirements for treatment to take place with minimal aesthetic impact. Thus, orthodontic treatment with lingual appliances meets the aesthetic requirements for a treatment that is as invisible as possible, while also being comfortable and providing the predicted final results [6].

The Invisalign^®^ System has contributed to meeting the aesthetic standards of patients during orthodontic treatment. Thus, the Invisalign^®^ System ClinCheck 6 can be successfully used in patients, especially for mild to moderate malocclusions that do not require tooth extractions. The introduction of new materials, i.e., Smart Force and Smart Track, in the Invisalign^®^ System has led to better control over tooth movements and an increase in the effectiveness of orthodontic treatment. These materials allow aligners to be designed so that they exert precise, constant, and directed forces on the teeth individually according to the needs of each patient. At the same time, orthodontic treatment can be made more efficient through digital planning with the help of ClinCheck^®^ 6 software [7].

Vestibular techniques of fixed orthodontic treatment using physiognomic materials have become more and more in demand in recent times, as they ensure satisfaction regarding the aesthetic standards of patients [8].

Orthodontic arches are basic components of orthodontic appliances. They play an essential role in orthodontic treatment, causing the teeth to move in the desired direction [6,8]. Currently, springs are made of different alloys. The analysis of the mechanical properties of the materials from which the arch is made is essential for the success of the orthodontic treatment and for the physiognomic requirements of the patient. The use of robotic systems in archbending represents a significant innovation in orthodontics, thus reducing errors. The emergence of new materials such as organic polymer wire or bactericidal springs has led to an improvement in patient comfort and aesthetics, a reduction in the risk of complications and infections, and the optimization of orthodontic treatment [9].

Along with the other techniques, orthodontic treatment with lingual appliances also meets the aesthetic requirements for a treatment that is as invisible as possible while being as comfortable as possible for the patient and providing predictable final results [10].

The purpose of this study is to determine the mechanical behavior of an orthodontic system where the lingual technique was used, in a real patient, from whom a set of CBCT scans was obtained. This set of tomographic images was processed by software specialized in recognizing the geometry of different tissues by identifying the range of shades of gray. Initially, a geometry composed of a so-called “point cloud” was obtained. This file contains a geometric structure similar to those obtained through three-dimensional scanning, and it has been subjected to specific reverse engineering techniques and methods [5].

In two-dimensional analyses of orthodontic treatments, not many aspects can be determined [11]. For this reason, in our study, we applied virtual techniques. 

The null hypothesis of the study is that, in the lingual technique, the forces that act on each element of the bracket type, and implicitly, the forces that act on each tooth, are equal because of the deformation of the orthodontic arch. 

This study has a strong instrumental aspect. Based on a model from a patient to whom a lingual orthodontic technique was applied, we obtained the mechanical behavior resulting from forces introduced by orthodontic arches using techniques and methods taken from direct engineering, reverse engineering, and the finite element method.

This study is significant because it will add more information to the literature regarding the forces generated on teeth subjected to the action of an orthodontic appliance applied through a lingual technique and will provide clinicians with evidence-based recommendations. Ultimately, this may lead to improved clinical outcomes in patients diagnosed with Angle Class I malocclusion dento-alveolar disharmony with crowding and a higher success rate for orthodontic treatment using the lingual technique.

## 2. Materials and Methods

The study was carried out in the Orthodontics Clinic of the Faculty of Dental Medicine Craiova. The Ethics Committee of the University of Medicine and Pharmacy Craiova (No. 152/11.07.2022) approved the conduct of this study. In accordance with the ethical guidelines for research with human participants of the University of Medicine and Pharmacy of Craiova, Romania, written informed consent was obtained from the legal guardians of the subjects involved in this study.

During 2023, 71 patients with Angle Class I malocclusion dento-alveolar disharmony with miner crowding were treated in the clinic. For 5 of them (7.04%), we chose the lingual technique as the method of orthodontic treatment. This study was conducted on a single patient who gave consent in this regard. Research on a significant group of patients was excluded because such an analysis requires significant software, hardware consumption, and financial resources. 

The inclusion criteria included patients with Angle Class I malocclusion dento-alveolar disharmony with miner crowding. 

The exclusion criteria included patients with Angle Class II malocclusion and Angle Class III malocclusion.

### 2.1. Examination of the Patient in Order to Establish the Diagnosis and Orthodontic Treatment Plan

**Figure 1:**
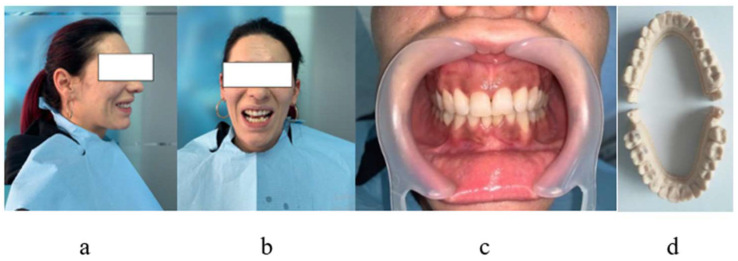
Patient photographs: (**a**) right profile; (**b**) front with open mouth; (**c**) endo-oral frontal photograph in occlusion; and (**d**) the study model.

A 25-year-old patient presented herself on 1 October 2023 at the Orthodontic Clinic of U.M.F Craiova for an orthodontic treatment. To establish the diagnosis, the patient was photographed as follows: the extraoral right profile, frontal with open mouth, and endo-oral frontal in occlusion. After the alginate impression of the patient’s dental arches, we obtained the study model. This study model was scanned in the dental laboratory. Figure 1a–d shows the images of the patient and of the study model. 

**Figure 2:**
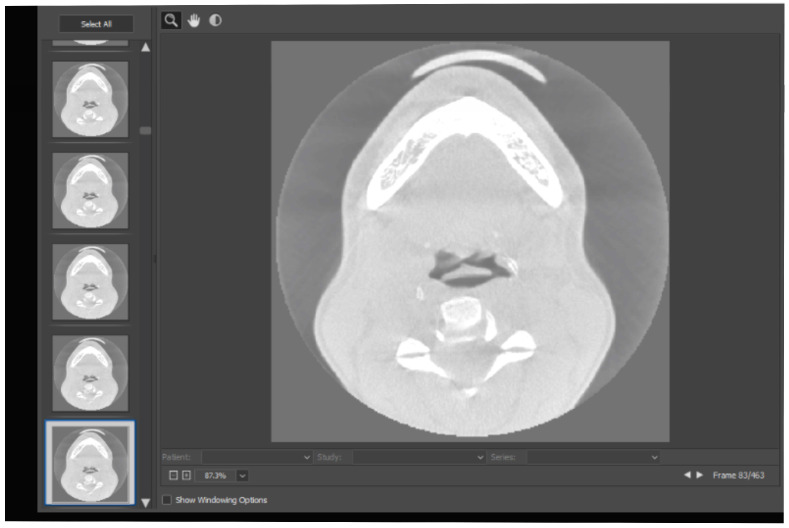
CBCT images of the analyzed patient.

The study model was obtained through rapid printing (3D) after dental scanning. Additionally, CBCT investigations were performed to ensure the accuracy of the diagnosis. Figure 2 shows the CBCT images of the analyzed patient.

Following clinical and paraclinical examination, the patient was diagnosed with Angle Class I malocclusion dento-alveolar disharmony with crowding.

For treatment, the patient was fitted with a fixed metal orthodontic appliance using the lingual technique. We used medium twin 2D brackets and two orthodontic archwires (maxillary and mandibular) with a round cross-section diameter of 0.012 inches (0.3048 mm) of Shape 3 type.

### 2.2. Application of the Finite Element Method

The computerized data analysis, three-dimensional modeling, and simulations using the finite element method were performed on multiple desktop computers (INTEL Core i3 processor with a frequency of 3.7 GHz, 466 GB hard disk, 8 GB RAM, Windows 10 64-bit operating system) and a Lenovo laptop system (INTEL Core i5 processor with frequency of 2.9 GHz, 476 GB SSD hard disk, 930 GB hard disk, 16 GB RAM, Windows 10 64-bit operating system).

To analyze the models and interpret the results, models, and result maps, a Legamaster smart board connected to a desktop computer with an i3 processor and an Optoma projector was used.

Through the Invesalius 3.1 program, CBCT images of tissues were transformed using special filters based on different shades of gray in these images. The results obtained through this program are similar to those obtained through three-dimensional scanning and are represented by the so-called “point cloud” [7].

Using the Geomagic Wrap 2019 program (which employs techniques and methods of reverse engineering), the point cloud was transformed into primary triangular surfaces. Subsequently, these surfaces were processed and converted into perfectly closed surfaces, which were then morphed into virtual solids [9,12].

Through the SolidWorks 2022 program (CAD—computer-aided design software), the perfectly closed surfaces were transformed into virtual solids. Thus, models containing virtual solids were created. With this program, we also modeled orthodontic arches and brackets [13,14].

Using the Ansys Workbench 2019 R3 program (software operating with the finite element method), these models were imported, and through successive simulations, displacement maps, deformations, stresses, and various diagrams were created [15,16,17,18].

From this suite of programs, Microsoft Excel 2019 was used extensively for calculations, data organization, and the application of special mathematical formulas, as well as for obtaining original graphs or diagrams.

This method, which, from the beginning, has a strong computational character, is based on the division of bodies and structures into finite elements, eventually linked by different connections and constraints and subjected to various loads or fields. These finite elements are three-dimensional geometric volumes that have sides, faces, and vertices called nodes. The equations that keep these elements linked in a body are called linkage equations. There are also equations that describe the behavior of forces or fields. At the same time, these equations can also contain unknowns. All these equations contain, in reality, differentials and complex derivatives. Even if the number of unknowns is equal to the number of equations, this system cannot be solved except in particular situations. However, this method contains a technique for simplifying differential equations and transforming them into linear equations. However, a system of linear equations with n equations and n unknowns can be solved using algorithms based on matrix calculation, regardless of how large the number n is. The results of applying the techniques of the finite element method are represented in maps and diagrams [19,20,21,22,23,24,25,26,27,28,29,30,31,32].

**Figure 3:**
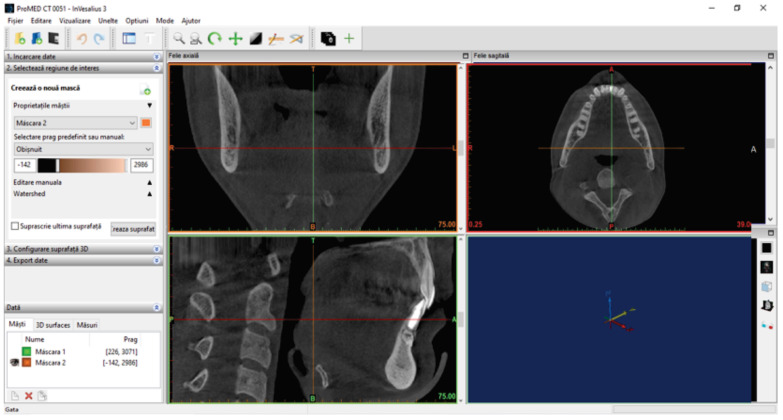
The initial interface of the Invesalius 3.1 program after loading the tomographies.

The set of CBCT scans of the patient was loaded into the Invesalius program, as shown in Figure 3. 

**Figure 4:**
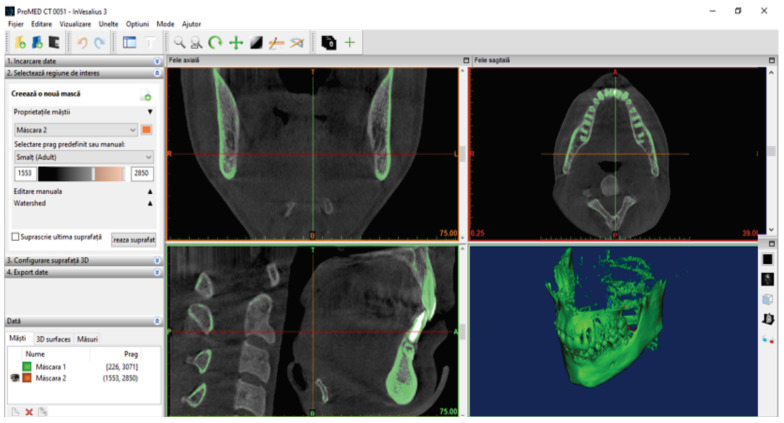
Using the enamel filter.

Initially, a filter for enamel was used, resulting in a new “point cloud”, as shown in Figure 4.

**Figure 5:**
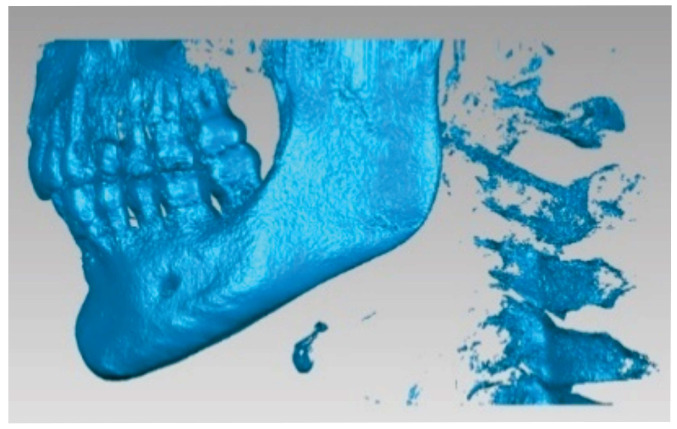
The analyzed model loaded into Geomagic.

Because the densities of enamel and bone components are relatively close, this model was loaded into the Geomagic X program for processing, as shown in Figure 5. Through processing, the primary geometry was transformed into triangular surfaces. Initially, the model contained 1.818.276 such surfaces.

**Figure 6:**
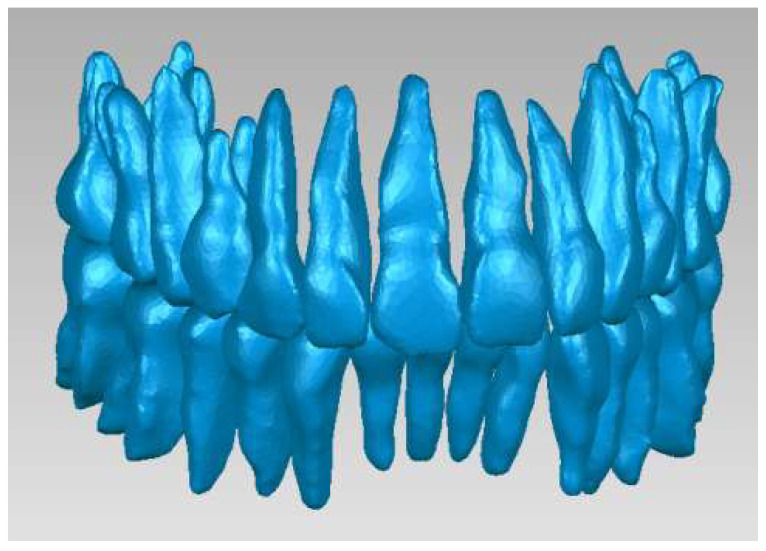
The final geometry in Geomagic.

Furthermore, specific techniques were used to remove portions of the mandible and maxilla. With the same techniques, we removed the remaining elements, and after several specific operations of reverse engineering, we obtained the final model of dental geometry, as shown in Figure 6.

**Figure 7:**
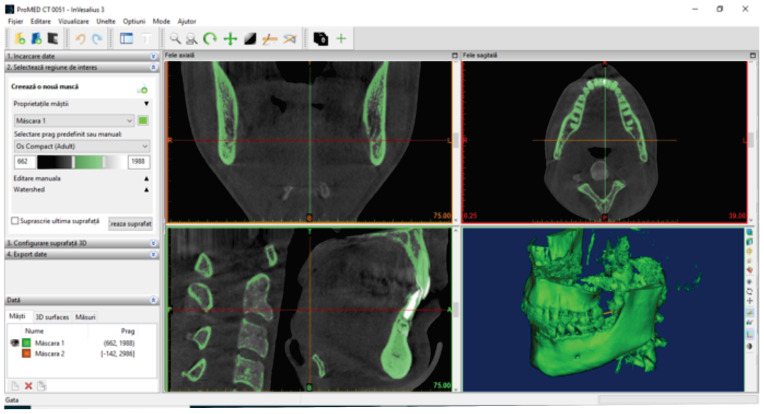
The initial geometry of the maxilla and mandible in Invesalius.

To determine the geometry of the two bone components (mandible and maxilla), we loaded the patient’s CBCT set into the Invesalius program. We used the filter for compact bone (adult) and the initial geometry. Figure 7 shows the initial geometry of the maxilla and mandible in Invesalius 3.1 [2].

**Figure 8:**
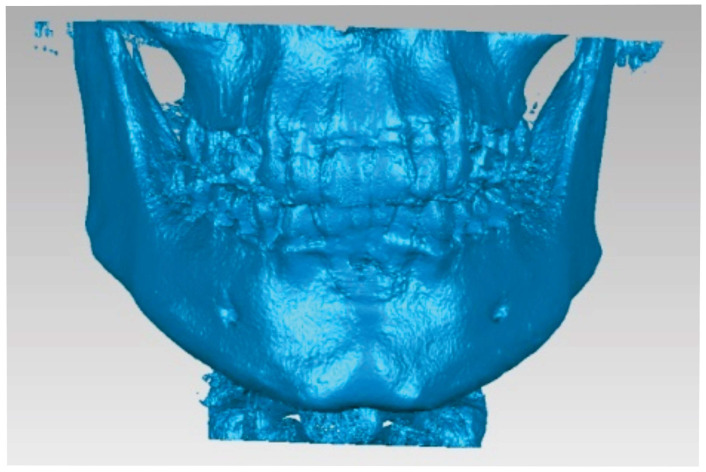
The initial model of the bone components in Geomagic.

Initially, the geometry of the two bone components was loaded into Geomagic for adaptation and processing (Figure 8).

The model, existing in Invesalius in the form of a “point cloud”, was automatically transformed into 2.914.308 primary triangular surfaces. In this study, only the fragments of the mandible and maxilla are of interest; therefore, the cervical spine and dental structure, previously determined, were removed from this model. Additionally, the model underwent operations to “fill” open surfaces, “decimate” the very large number of elementary triangular surfaces, perform “finishing” operations, and eliminate non-conforming elementary surfaces.

**Figure 9:**
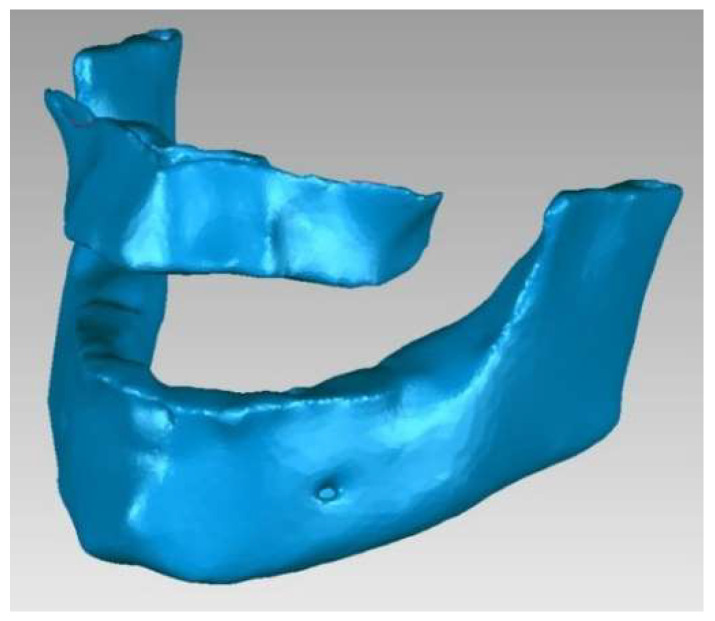
The final model of the two bone components.

In the end, the model of the two bone components consisted of two perfectly closed surfaces and comprised 56.296 elementary triangular surfaces (Figure 9).

**Figure 10:**
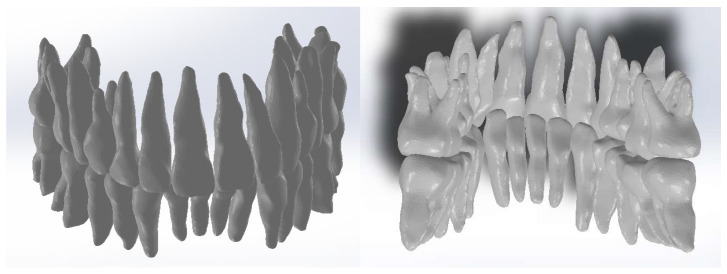
The tooth models transformed into virtual solids.

Continuing, we loaded the model of the teeth into the SolidWorks program, one by one, and then the model of the two bone components. In Geomagic, the tooth model composed only of perfectly closed surfaces was obtained without non-conforming surfaces. In SolidWorks, the models were automatically converted into virtual solids, as shown in Figure 10.

Additionally, the models of the two bone components (mandible and maxilla) were also loaded into SolidWorks and automatically converted into virtual solids.

**Figure 11:**
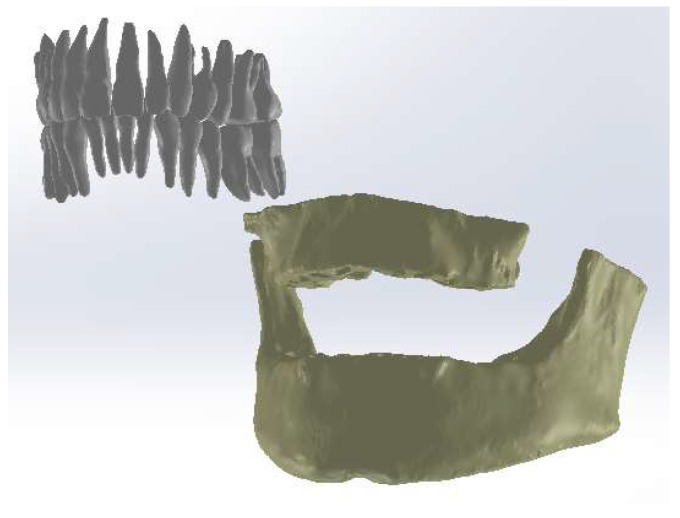
The two models in the Assembly module of SolidWorks.

Continuing, we loaded the two models into the Assembly module of the SolidWorks program, as shown in Figure 11.

**Figure 12:**
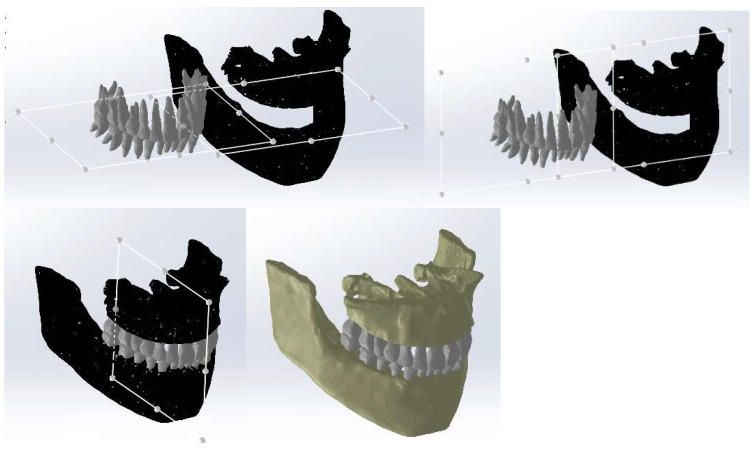
Positioning the models of bone components and teeth.

For the correct positioning of the two models, we used reference planes that should coincide because both models originate from a single set of CBCT scans, as shown in Figure 12.

**Figure 13:**
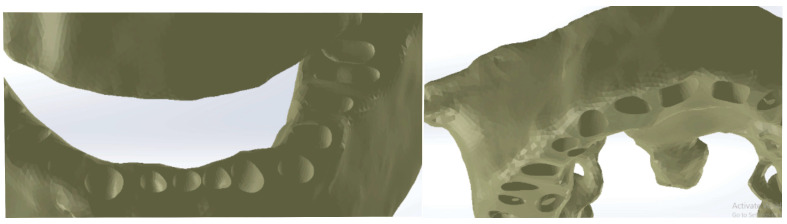
Generating the tooth sockets by subtracting the volume.

Next, the tooth sockets were created by subtracting the volume of the tooth models from the models of bone components (using the Cavity function in SolidWorks 2022 Assembly), as shown in Figure 13.

**Figure 14:**
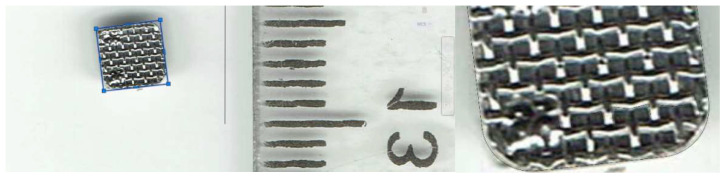
Drawing the main outline of the bracket component.

In parallel, models for orthodontic archwire and bracket were generated. To determine the model of a bracket, initially, a photographic image was obtained next to a ruler graduated in millimeters. The images were imported into the AutoCAD 24.0 program, which is specialized for flat drawing. In this software, the main outline of the bracket component was obtained (Figure 14).

**Figure 15:**
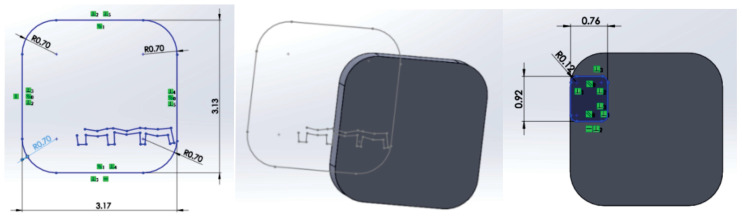
Defining the base of the bracket component in SolidWorks.

The outline of the bracket component was imported into SolidWorks 2022, where modeling began using CAD techniques and methods of direct engineering (Figure 15). 

**Figure 16:**
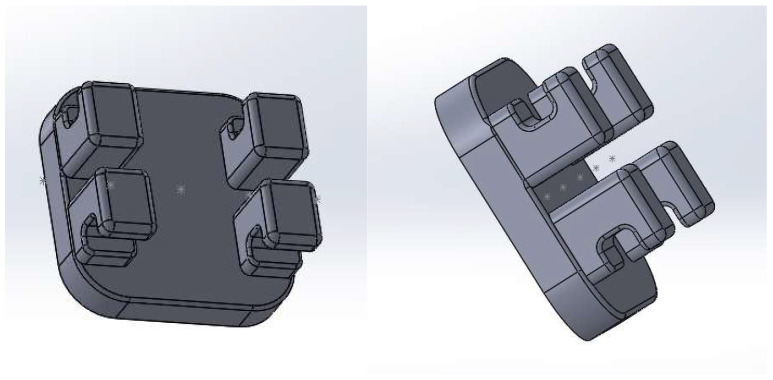
The model of the bracket components.

In the end, with the help of several three-dimensional modeling techniques, the final model of the bracket component was obtained. Additionally, 5 points were defined at a distance greater than 0.012 inches (0.3 mm) from the base of the bracket component ears, as shown in Figure 16. These points were necessary for the correct guidance of the orthodontic archwire during assembly and when defining its main curve [23,24,25,26,27,28,29,30,31,32,33,34,35].

**Figure 17:**
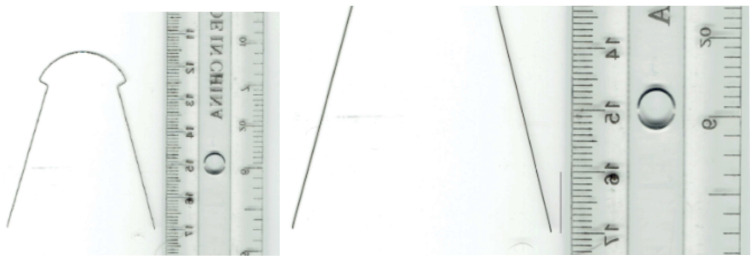
Determining the main curve of the orthodontic archwire.

To obtain the model of the undeformed orthodontic archwire, it was scanned two-dimensionally next to a graduated ruler. The image was loaded into AutoCAD where it was scaled to the natural scale 1:1. In AutoCAD, the main curve defining the undeformed orthodontic archwire was obtained (Figure 17).

**Figure 18:**
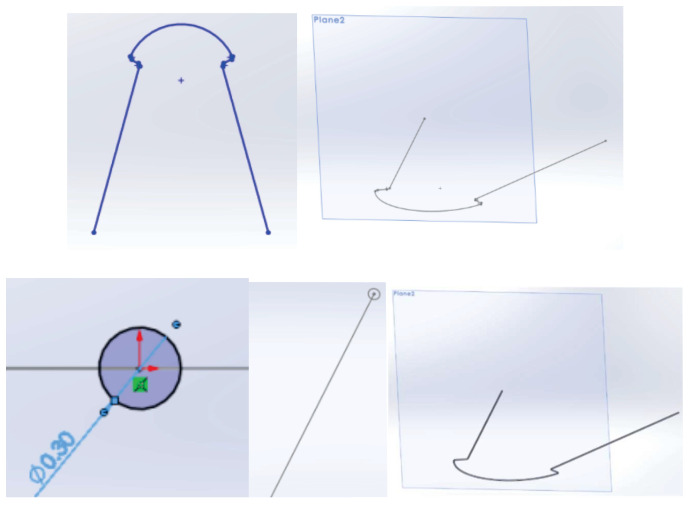
Modeling steps of the undeformed orthodontic archwire.

This contour was imported into SolidWorks on a flat sketch. Then, a plane perpendicular to the flat curve was defined, with the origin on the curve. In this plane, a circle was sketched with the center at the origin. Finally, the virtual solid that unifies the trajectory (flat curve) and the circle with a diameter of 0.012 inches was defined, as shown in Figure 18.

Next, we virtually placed the bracket components on the orthodontic model, which, at that point, contained the tooth models and bone components. To make this step easier, initially, the bone components were temporarily suspended. At this stage, the mounting protocols in lingual technique were taken into account.

**Figure 19:**
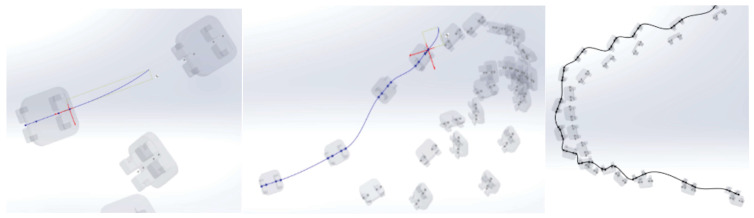
Defining the spatial curve that defines the orthodontic archwire for the maxillary teeth.

To generate the models of the two orthodontic archwires, initially, both the teeth and bone components were temporarily suppressed. To define the guiding curve (trajectory) of an orthodontic archwire, a spatial curve passing through all 5 characteristic points of the bracket components was defined (Figure 19).

**Figure 20:**
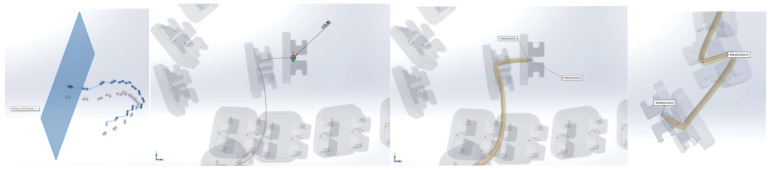
Generating an orthodontic archwire.

Next, a plane perpendicular to the curve was defined with the origin at one end. In this plane, a circle with a diameter of 0.012 inches was drawn. These two curves defined the virtual solid of the orthodontic archwire (Figure 20).

**Figure 21:**
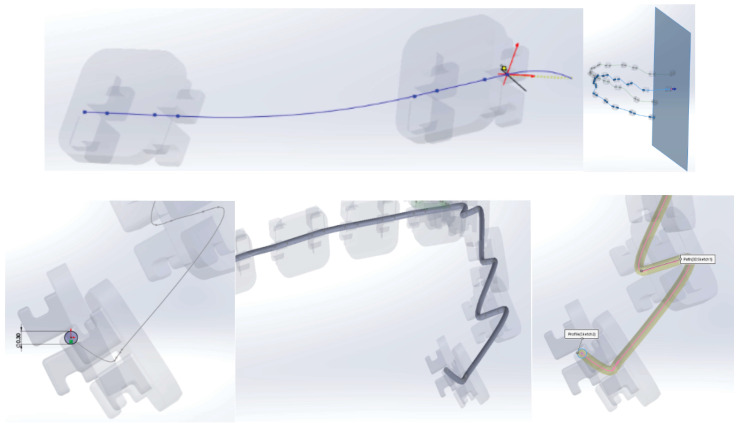
Defining the orthodontic arch for the lower mandibular teeth.

Similarly, we proceeded to the other orthodontic archwire (Figure 21).

**Figure 22:**
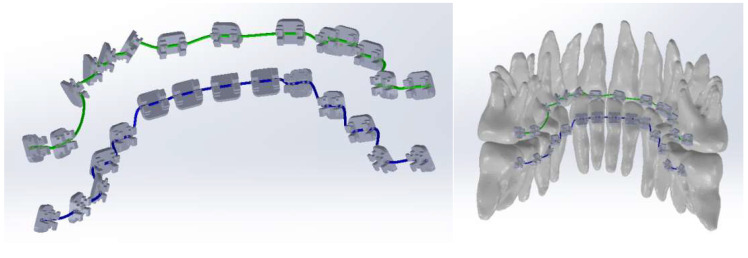
The models of the two orthodontic archwires.

**Figure 23:**
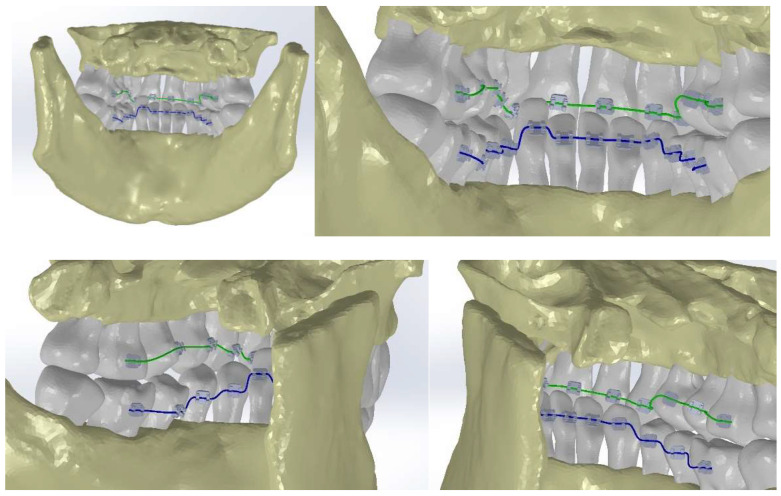
The final model of the analyzed orthodontic system.

In Figure 22, the two orthodontic archwires are presented, one for the maxilla in green and one for the mandible in blue. In Figure 23, the final model of the analyzed orthodontic system is presented.

## 3. Results

### 3.1. Analysis of CBCT Images in Order to Choose the Orthodontic Treatment Plan

**Figure 24:**
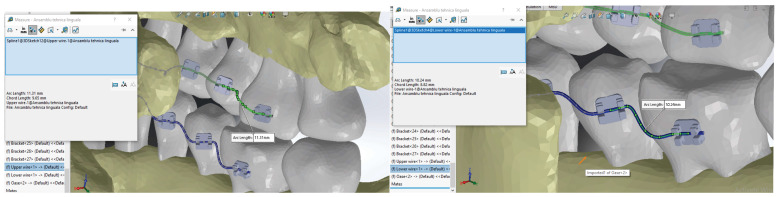
Measuring the distances between teeth on the orthodontic archwire curve.

The curves defining the orthodontic archwires are actually a chain of spatial Spline curves with ends on the bracket components. These can be directly measured in SolidWorks using the Measure function (Figure 24).

For the maxilla, the distances between adjacent teeth measured on the orthodontic archwire curve are presented in Table 1, and for the mandible, they are presented in Table 2.

### 3.2. Finite Element Analysis of the Forces Generated by the Application of an Orthodontic Treatment Method Using the Lingual Technique

To determine the maximum deformations of the orthodontic archwires for each tooth, we calculated the lengths used from the initial orthodontic archwire by summing the values in Table 1 and Table 2. For the maxilla, we determined a length of 89.14 mm, and for the mandible, a length of 75.67 mm.

**Figure 25:**
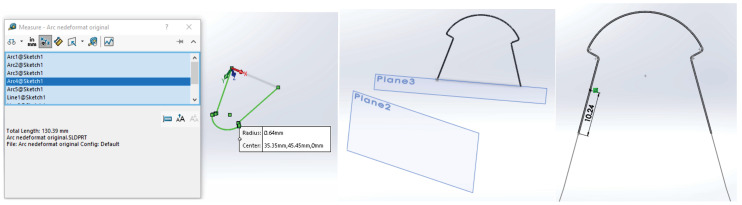
Determining the initial length and adjustment of the orthodontic archwire.

Additionally, we directly measured the initial length of the undeformed orthodontic archwire in SolidWorks, resulting in a length of 130 mm. These lengths revealed that the maxillary arch was shortened by 44.57 mm, while the mandibular arch was shortened by 37.83 mm. Taking these determinations into account, the used arches were adjusted to the determined dimensions (Figure 25).

Next, using the values in Table 1 and Table 2, we marked the positions of the teeth by points on the undeformed orthodontic archwire. Then, in the Assembly module of SolidWorks, we overlaid the two models, the deformed one and the undeformed one.

Having marked the positions of the teeth on the two models, we drew straight line segments spatially connecting the corresponding positions of the teeth.

**Figure 26:**
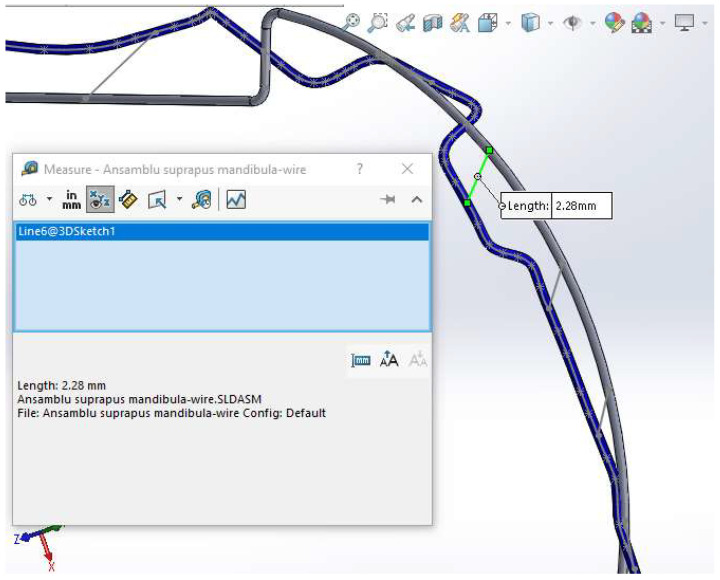
Measuring the distances of deformation on the orthodontic archwire for each tooth.

These segments were virtually measured, representing the extent of deformations occurring on the orthodontics archwire for each tooth (Figure 26).

These data, representing the maximum deformations in the orthodontic archwire, are synthesized and organized in Table 3 for the maxilla and in Table 4 for the mandible.

Next, we determined the elastic forces acting on each tooth in the patient treated with the lingual orthodontic technique. To determine the forces acting on the marginal teeth (16, 26, 46, and 36) of each tooth, Formula (1) was analyzed:

(1) $$F=\frac{3\cdot E\cdot I\cdot s}{L^{3}},$$

To determine the forces on the marginal teeth relative to the orthodontic archwires, Formula (1) becomes:

(2) $$F_{ij}=\frac{3\cdot E\cdot I\cdot s_{ij}}{{L_{ij}}^{3}}$$

where *i* = 1 … 4; *j* = 6; *s* = the maximum deformation of the orthodontic archwire, which is listed in Table 3 and Table 4 (i.e., S16, S26, S46, and S36); *F* = the force acting on the tooth trough the corresponding bracket; *L* = the distance measured on the orthodontic archwire curve, which is listed in Table 1 and Table 2 (i.e., L16-15, L25-26, L45-46, and L35-36); *E* = the modulus of elasticity (Young’s modulus), where E = 3.45 × 1010 for Nitinol; and I = the moment of inertia, and in the case of a round cross-section bar, this is $=\frac{\pi \cdot d^{4}}{64}$, where *d* is the diameter of the orthodontic archwire, d = 0.012 inch, d = 0.3048 mm.

After performing the calculation, I = 3.975 · 10^−16^ m^4^.

In addition, for the other teeth, the force is determined by the formula:

(3) $$F=\frac{9\;\sqrt{3}\cdot L\cdot E\cdot I\cdot s}{a\cdot {\left(L^{2}-a^{2}\right)}^{3/2}}$$

Adapting the formula for the analyzed situation, it becomes:

(4) $$F_{ij}=\frac{9\;\sqrt{3}\cdot \left(L_{i,j-1}+L_{i,j-2}\right)\cdot E\cdot I\cdot s_{ij}}{a\cdot [{\left(L_{i,j-1}+L_{i,j-2}{)}^{2}-{L_{ij}}^{2}\right]}^{3/2}}$$

where i = 1 … 4; j = 5 … 3.

Applying these formulas yields the values for the forces F, as shown in Table 5 and Table 6.

To determine the mechanical behavior of the studied orthodontic system, we used the finite element method. The model of the lingual orthodontic system was imported into Ansys Workbench. 

To obtain an accurate solution, the models of the two orthodontic archwires were suppressed and replaced with the forces determined through analytical methods in the previous subsection. Additionally, the physical and mechanical properties of the study components were added to the Ansys program database (Table 7) [36,37].

**Figure 27:**
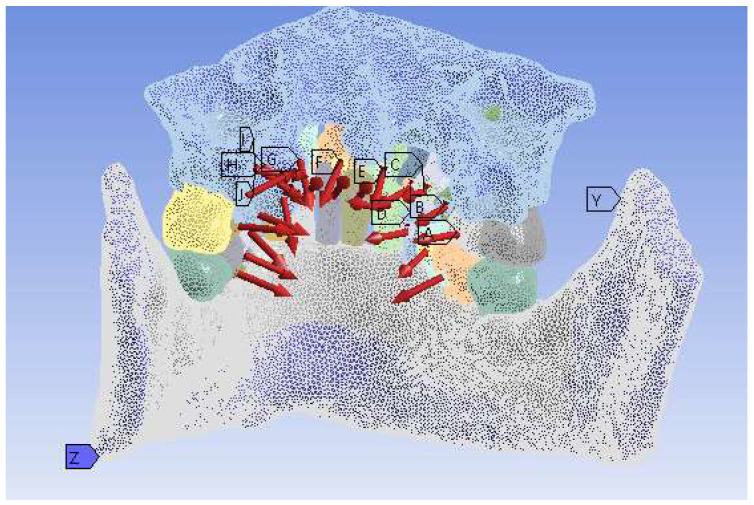
The force system applied to the analyzed system. The letters in the image were created automatically by the software in the order of defining the forces.

Next, the model was divided into tetrahedral finite elements. The two bone components were considered fixed. The force system is shown in Figure 27. The force values are those obtained earlier through analytical methods.

**Figure 28:**
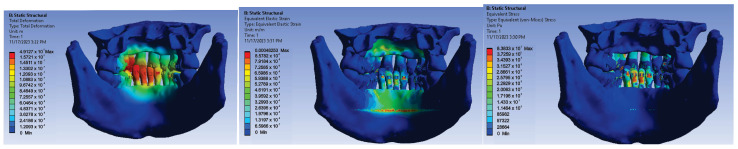
(**a**) Displacement map; (**b**) deformation map; and (**c**) stress map.

After running the simulation, maps of results including displacements, deformations, and stresses were obtained based on the von Mises criterion (Figure 28a–c).

Analyzing the values of the forces resulting from the elasticity of the orthodontic archwires, we found that in the lingual technique, the smallest force appearing on the upper maxillary teeth was 0.1239 N and was located at tooth 1.1. The greatest force at the upper maxilla level occurred at 2.3 and had a value of 1.568 N. In the mandible, the smallest force appeared at 4.6, while the largest force was at 3.1 with a value of 1.493 N. Examining the result maps obtained through FEM simulation, in the lingual technique, the maximum value of displacements in the analyzed orthodontic system was 9127 × $10^{7}$ m, of the deformations was 0.00048253, and of mechanical stresses was 8.3833 × $10^{7}$ Pa.

## 4. Discussion

The null hypothesis of this study is that, in the lingual technique, the forces that act on each element of the bracket type, and implicitly, the forces that act on each tooth, are equal. The results of our study reject the null hypothesis. In the lingual technique, the forces that act on each element of the bracket type, and implicitly, the forces that act on each tooth, are not equal because of the deformation of the orthodontic arch.

These changes in the forces generated by orthodontic treatment through the lingual technique have clinical significance and allow for obtaining predictive results.

In recent years, three-dimensional reconstruction methods using CBCT images have become increasingly prevalent in dental research. These methods transform two-dimensional images into “point cloud” structures, similar to three-dimensional scanning but much deeper, capturing various internal structures and pathologies [12,38].

Engineering techniques and methods have gained significant importance in dentistry, especially in recent years. Inverse engineering methods allow for the analysis of tissue structures and the primary three-dimensional modeling thereof, forming the basis for various virtual simulations. These techniques and methods result in obtaining three-dimensional structures similar to those found in the patient’s body [39,40].

The finite element method can be successfully used not only in orthodontics but also in prosthetics. The finite element method can be essential in studying the effects of the materials used in making Zirconium implants around the cortical bone of dental prostheses, thus improving the quality of the treatment [41].

Thus, with the help of the finite element method, one study investigated the behavior of a fixed prosthesis system OT Bridge (Rhein 83^®^). The finite element method had an important role in understanding and managing the stresses that appear in fixed OT Bridge prostheses anchored on implants [42].

We can also use the finite element method in orthodontics. In our case, three-dimensional reconstruction techniques were applied to a set of CBCT scans taken from a patient with Angle Class I malocclusion dento-alveolar disharmony with miner crowding. The dental geometry was reconstructed and, using CAD techniques, orthodontic-specific components were added to the model, such as the bracket system and the two orthodontic archwires used in the lingual technique. Once this model, similar to the patient’s structures, was created, we analyzed the lingual technique used in orthodontics.

Experimental determination of the forces occurring in such an orthodontic system is practically impossible. We chose a non-invasive and non-destructive method, i.e., the finite element method, after applying different medical imaging and reverse engineering techniques that brought the model of the real patient into a virtual analysis environment. Using methods and techniques found in materials science and elasticity theory, we determined the forces acting on each tooth.

Once these forces were determined, we used them in the loading system on the model imported into Ansys Workbench, a program that utilizes the finite element method. This method is non-invasive and non-destructive and is increasingly used in the medical field. The method involves dividing structures into small volumes (finite elements) to which linear systems of equations derived from differential equations are applied, depicting the mechanical behaviors of systems. The results obtained through such simulations consist of diagrams and result maps that express the behavior of the analyzed orthodontic system.

The force intensity values occurring during treatment with the lingual technique, determined in this study, are similar to those in other studies. This study, based on virtual techniques, demonstrates that forces acting on each tooth of the orthodontic system, similar to those used in real patients treated with the lingual technique, can be calculated. The finite element method allows for the determination of displacements, deformations, and stresses [43,44].

Regarding pain, psychological discomfort, and social disability, one study found that patients with a lingual bracket showed the least amount of pain at all times and the least detrimental effects. No significant differences were found between patients with conventional, low-friction, lingual brackets and aligners in terms of quality of life [45]. 

Numerous research studies have looked into patients’ attitudes and oral discomfort following the insertion of lingual appliances [46,47].

Currently, systems exist that use computer-aided design (CAD) and computer-aided manufacturing (CAM) to build lingual brackets. Because of the fact that this technology combines specialized metal pads and brackets into a single unit, patients experience less discomfort [48]. Even with the advances in technology made possible by 3D digitalization, lingual appliances remain expensive when compared with more traditional lingual techniques [49].

Recent research has paid special attention to the measurement of the intensity of the occlusion force, which has become one of the important markers in the evaluation of the long-term stability of orthodontic treatment. Periodic evaluation of the intensity of the occlusion force during orthodontic treatment allows the doctor to intervene by modifying the occlusion to ensure the success of the orthodontic treatment and the patient’s comfort. Currently, several devices have been created to measure the intensity of the occlusion force. They may be modified in the future to ensure better accuracy in the diagnosis of dento-maxillary anomalies and implicit orthodontic treatment. Thus, strain-gauge transducers (Flexiforce and T-scan), by reducing the thickness of the sensor and improving its precision, will be able to easily measure the intensity of the occlusion force [50].

The forces exerted on the teeth during orthodontic treatment can sometimes cause orthodontic-induced inflammatory root resorption (OIIRR). The cause of orthodontic-induced inflammatory root resorption (OIIRR) is the body’s immune response to the action of orthodontic forces. Thus, the use of transgenic/gene knockout technologies could be extremely useful to better understand the cellular and molecular immunological mechanisms involved in the occurrence of orthodontically induced inflammatory root resorption (OIIRR) [51].

Under the action of orthodontic forces, orthodontic tooth movement (OTM—orthodontic tooth movement) occurs through bone remodeling. With the help of osteoimmunology, the correlation between bone and soft tissue remodeling and the body’s immune reaction caused by OTM is analyzed. Thus, by understanding the mechanisms of OTM, orthodontic practice can be improved by the emergence of faster, more efficient, and more comfortable treatments with fewer side effects for patients [52].

The finite element method (FEM) is a valuable tool in orthodontic research because it highlights a number of important points, including the following: the direction of tooth displacement; the optimal location of orthodontic appliances during a particular mechanics; and the zones most likely to exhibit root resorption [53].

The novelty of this study is that it was carried out on a real case, where, through virtual techniques, the real geometries of the patient’s tissues were brought into a virtual environment. For analysis and study, we detailed the techniques and methods that allow for determining the forces acting on such a system, as well as the effects produced by them. 

The clinical significance of this study is that the results have important implications for the choice of orthodontic arches, bracket-type elements, and specific orthodontic methods. 

### 4.1. Limitations of This Study

The limitations of this study should be carefully highlighted when interpreting the results. First, this study only included a finite element analysis, which means the results may not accurately reflect what happens in the clinical setting.

While this study provides valuable information regarding the forces generated during orthodontic treatment using the lingual technique, its limitations suggest that further research is needed to determine the safety of the results obtained with this method of orthodontic treatment.

In the specialized literature, we did not find similar studies carried out on real patients. This study verifies the lingual orthodontic technique in a virtual space by determining the forces that appear in this system and also their effects, such as displacement, strain, and stress maps, which can give us information about the mechanical behavior of the system we analyzed.

### 4.2. Recommendations for Further Research

Comparison of the results obtained from our study with other future studies on patients with similar pathology is essential to validate and generalize our conclusions. Other virtual methods and techniques can be used in future studies, including the application of virtual reality for orthodontic diagnosis.

## 5. Conclusions

The values of the forces and the results of the finite element analysis represent a basis for the innovation of new orthodontic springs and bracket elements. Thus, by using new technologies, orthodontic practice can be significantly improved for the benefit of patients.

## Figures and Tables

**Table 1:** Distances between teeth measured on the orthodontic archwire curve for the maxilla.

Distances between Teeth Measured on the Orthodontic Archwire Curve for the Maxilla [mm]										
L16-15	L15-14	L14-13	L13-12	L12-11	L11-21	L21-22	L22-23	L23-24	L24-25	L25-26
11.31	7.07	10.77	6.6	7.54	7.18	5.66	6.95	5.66	8.24	12.16

**Table 2:** Distances between teeth measured on the orthodontic archwire curve for the mandible.

Distances between Teeth Measured on the Orthodontic Archwire Curve for the Mandible [mm]										
L46-45	L45-44	L44-43	L43-42	L42-41	L41-31	L31-32	L32-33	L33-34	L34-35	L35-36
10.24	8.07	7.19	5.33	4.57	4.47	4.45	7.07	7.16	6.92	10.2

**Table 3:** Maximum deformations in the orthodontic archwire for the maxilla.

Maximum Deformations in the Orthodontic Archwire for the Maxilla [mm]											
S16	S15	S14	S13	S12	S11	S21	S22	S23	S24	S25	S26
6.39	5.75	4.31	2.19	1.45	0.6	0.97	3.62	4.71	4.09	5.05	5.5

**Table 4:** Maximum deformations in the orthodontic archwire for the mandible.

Maximum Deformations in the Orthodontic Archwire for the Mandible [mm]											
S46	S45	S44	S43	S42	S41	S31	S32	S33	S34	S35	S36
2.53	4.2	3.73	0.85	1.61	1.62	1.61	2.28	1.99	1.55	3.17	3.11

**Table 5:** The forces on the bracket elements placed on the maxilla.

The Forces on the Bracket Elements—Maxilla [*N*]											
F16	F15	F14	F13	F12	F11	F21	F22	F23	F24	F25	F26
0.181	0.656	0.529	0.298	0.339	0.123	0.307	1.205	1.568	1.049	0.411	0.125

**Table 6:** The forces on the bracket elements placed on the mandible.

The Forces on the Bracket Elements—Mandible [*N*]											
F46	F45	F44	F43	F42	F41	F31	F32	F33	F34	F35	F36
0.096	0.459	0.694	0.293	1.101	1.443	1.493	1.051	0.454	0.365	0.436	0.12

**Table 7:** Physical and mechanical properties of the study components.

Component	Material	Density	Young’s Modulus	Transverse Elasticity Modulus	Poisson’s Ratio
Orthodontic archwire	Nitinol	6.450 kg/m^3^	8.3 × 10^7^ Pa	3.12 · 10^7^ Pa	0.33
Bracket type elements	Alloy Ni + Cr	8.500 kg/m^3^	2.1 × 10^11^ Pa	8.015 · 10^10^ Pa	0.31
Maxilla, mandible	Bone	1.400 kg/m^3^	1 × 10^10^ Pa	3.84 · 10^9^ Pa	0.31
Teeth	Enamel	2.958 kg/m^3^	7.79 ×·10^10^ Pa	2.996 · 10^10^ Pa	0.3

## Data Availability

The authors declare that the data from this research are available from the corresponding authors upon reasonable request.
